# A Cancer Associated Fibroblasts-Related Six-Gene Panel for Anti-PD-1 Therapy in Melanoma Driven by Weighted Correlation Network Analysis and Supervised Machine Learning

**DOI:** 10.3389/fmed.2022.880326

**Published:** 2022-04-11

**Authors:** Luyao Tian, Fei Long, Youjin Hao, Bo Li, Yinghong Li, Ying Tang, Jing Li, Qi Zhao, Juan Chen, Mingwei Liu

**Affiliations:** ^1^Key Laboratory of Clinical Laboratory Diagnostics, College of Laboratory Medicine, Chongqing Medical University, Chongqing, China; ^2^Cell Biology and Bioinformatics, College of Life Sciences, Chongqing Normal University, Chongqing, China; ^3^Key Laboratory on Big Data for Bio Intelligence, Chongqing University of Posts and Telecommunications, Chongqing, China

**Keywords:** melanoma, anti-PD-1 therapy, CAFs-related biomarker panel, WGCNA, supervised machine learning

## Abstract

**Background:**

Melanoma is a highly aggressive skin cancer with a poor prognosis and mortality. Immune checkpoint blockade (ICB) therapy (e.g., anti-PD-1 therapy) has opened a new horizon in melanoma treatment, but some patients present a non-responsive state. Cancer-associated fibroblasts (CAFs) make up the majority of stromal cells in the tumor microenvironment (TME) and have an important impact on the response to immunotherapy. There is still a lack of identification of CAFs-related predictors for anti-PD-1 therapy, although the establishment of immunotherapy biomarkers is well underway. This study aims to explore the potential CAFs-related gene panel for predicting the response to anti-PD-1 therapy in melanoma patients and elucidating their potential effect on TME.

**Methods:**

Three gene expression datasets from melanoma patients without anti-PD-1 treatment, in a total of 87 samples, were downloaded from Gene Expression Omnibus (GEO) as the discovery sets (GSE91061) and validation sets (GSE78220 and GSE122220). The CAFs-related module genes were identified from the discovery sets by weighted gene co-expression network analysis (WGCNA). Concurrently, we utilized differential gene analysis on the discovery set to obtain differentially expressed genes (DEGs). Then, CAFs-related key genes were screened with the intersection of CAFs-related module genes and DEGs, succeeded by supervised machine learning-based identification. As a consequence of expression analysis, gene set enrichment analysis, survival analysis, staging analysis, TME analysis, and correlation analysis, the multidimensional systematic characterizations of the key genes were uncovered. The diagnostic performance of the CAFs-related gene panel was assessed by receiver operating characteristic (ROC) curves in the validation sets. Eventually, the CAFs-related gene panel was verified by the expression from the single-cell analysis.

**Results:**

The six-gene panel associated with CAFs were finally identified for predicting the response to anti-PD-1 therapy, including *CDK14, SYNPO2, TCF4, GJA1, CPXM1*, and *TFPI*. The multigene panel demonstrated excellent combined diagnostic performance with the area under the curve of ROC reaching 90.5 and 75.4% ~100% in the discovery and validation sets, respectively.

**Conclusion:**

Confirmed by clinical treatment outcomes, the identified CAFs-related genes can be used as a promising biomarker panel for prediction to anti-PD-1 therapy response, which may serve as new immunotherapeutic targets to improve survival outcomes of melanoma patients.

## Introduction

Cutaneous melanoma is a highly aggressive malignancy that has been found to contribute to ~1.7% (232,100 cases) of all newly diagnosed primary malignant cancers, leading to a~55,500 deaths every year (1). Recent clinical studies promised the great advantages of anti-PD-1 immunotherapy over traditional treatments, including superior clinical efficacy and significant survival benefits for melanoma patients (2). Meanwhile, anti-PD-1 therapy for melanoma remains challenging because of its heterogeneous immune response of 30–60% of patients characterized by little or no response (3, 4). Moreover, immunotherapy is expensive and patients usually are involved in a high-grade immune-related adverse event (5). In terms of the clinical response, costs, and side effects, it is urgent to find biomarkers to predict the efficacy of anti-PD-1 therapy.

PD-L1 immunohistochemical assay is commonly used in predicting the efficacy of anti-PD-1 therapy. However, the variety of PD-L1 expression over time leads to its insufficiency as a routine clinical biomarker (6). Tumor mutation burden (TMB), microsatellite instability (MSI), and molecular subtypes can predict the clinical response of patients (7). Unfortunately, these detection ways are inconvenient and expensive because of complex molecular detection methods. Therefore, we necessary explore faster and more economical predictors for anti-PD-1 therapy response.

The tumor microenvironment (TME), a complex system composed of tumor cells, stromal cells, infiltrating immune cells, and abundant extracellular matrix (ECM), has a strong connection with tumor immunosuppression (8). Cancer-associated fibroblasts (CAFs) constitute vastly heterogeneous stromal cells and are prominent components of the microenvironment in solid tumors. To date, it has been recognized as one of the most promised biomarkers of immunosuppressive TME (9).

CAFs are the activated fibroblasts in the early stage of tumor development marked by the heterogeneity of origin, phenotype, and function. Studies identified CAFs promote the development of malignant tumors through various mechanisms, including secreting growth factors, remodeling the extracellular matrix, promoting angiogenesis, and mediating tumor-promoting inflammation. Furthermore, it is also involved in tumor immune escape by affecting immune cell infiltration, inducing immunosuppression, and inhibiting lymphocyte tumor-killing effects (10, 11). Studies demonstrated that CAFs can inhibit CD8^+^ T cells and activate FoxP3^+^ lymphocytes by secreting IL-6 and thus promote immunosuppression of TME (9). The tumor-associated macrophages (TAM) are part of inflammatory infiltrating cells in TME, of which the M2 type has immunosuppressive functions. CAFs have been proved to recruit monocytes and induce their differentiation into M2 type via monocyte chemotactic protein 1 (MCP-1) and chemokine CXCL-12 (12). Importantly, CAFs have been proved to directly reduce the activation of CD8^+^ T cells and natural killer (NK) cells by upregulating the expression of immune checkpoint signals (e.g., PD-1, PD-L1, PD-L2), which in turn hinders anti-tumor immunity (13, 14). In conclusion, CAFs can promote tumor immunosuppression and immune escape by interacting with the immune cell through the secretion of cytokines, chemokines, and other cytokines.

The evidence above suggests the association between CAF-related genes and the effectiveness of anti-PD-1 therapy in tumor patients. There are some studies foucusing on biomarkers for the response prediction of anti-PD-1 therapy in melanoma, but the majority of them tend to ignore the exploration of the functional correlation between genes as well as the regulatory mechanism of genes in TME. Furthermore, the exploration of anti-PD-1 therapy response biomarkers related to CAFs in melanoma is few up to now. Therefore, in this study, a robust analysis, comprised of weighted gene co-expression network, least absolute shrinkage and selection operator (LASSO), and random forest machine learning, was applied to screen CAFs-related gene panel associated with anti-PD-1 therapeutic response in melanoma, aiming to understand the potential functions of these genes in TME and evaluate their anti-PD-1 therapeutic response prediction performance.

Here, three existing RNA-seq datasets on anti-PD-1 immunotherapy of melanoma were analyzed using a bioinformatics approach. First, GSE91061 was applied to construct a gene co-expression network by weighted gene co-expression network analysis (WGCNA) to screen key modules related to CAFs score. Functional enrichment analysis was further to investigate the biological function of the key module genes. Second, six CAFs-related genes for predicting response to anti-PD-1 therapy were identified with the combination of LASSO and random forest. Subsequently, adopting gene set enrichment analysis (GSEA) and gene expression profiling interactive analysis (GEPIA), the biological functions and survival/staging associations of the six genes were explored based on TCGA-SKCM RNA sequencing data. To gain insight into the impact and association of these molecules on immune/stromal cells in TME, xCELL was applied to explore the correlation of the six genes with immune/stromal cells. Not only that, the multivariate Cox model confirmed the excellent performance of the six-gene panel in survival prediction, and TME analysis demonstrated significant TME alterations existed in different risk groups. Further gene correlation analysis revealed significant positive correlations of these six genes with anti-tumor immune-related molecules, immune checkpoint-associated molecules, and CAFs-related stimulators, which suggested the potential anti-tumor immune functions. After validating the prediction accuracy of the gene panel in the GSE78220 and GSE122220 datasets, we finally summarized key features of the KCDEGs and investigated single-cell localizations to determine their significant expression in CAFs and TME-associated cells.

## Methods

The workflow of this study is shown in Supplementary Figure 1, including three main parts, WGCNA-based CAFs-related differential gene screening, key gene extraction by machine learning models, and key gene validation based on multidimensional system analysis. The details are described as follows.

### Acquisition of Gene Expression Data

Datasets were collected using “melanoma,” “SKCM,” “PD-1,” “treatment,” “therapy” and “immunotherapy” as keywords in Gene Expression Omnibus (GEO) (http://www.ncbi.nlm.nih.gov/geo) (15), ArrayExpress (https://www.ebi.ac.uk/arrayexpress) (16) and Expression Atlas (https://www.ebi.ac.uk/gxa/home) (17). The collected data were reviewed and included by the following standards: (i) gene expression datasets included responsive (full response or partial response) or non-responsive (disease progression or disease stabilization) melanoma patients to anti-PD-1 therapy. Responders and non-responders to immunotherapy were confirmed consulting iRECIST guidelines (18); (ii) each dataset contained at least 10 samples; (iii) the data were publicly accessible and downloadable. Ultimately, three datasets (*n* = 87) were filtered out and downloaded from GEO, including GSE91061 (39 non-responders and 10 responders) (19), GSE78220 (13 non-responders and 15 responders) (20) and GSE122220 (6 non-responders and 4 responders). Additionally, GSE114445 was downloaded to explore gene expression in normal skin (*n* = 6), nevus (*n* = 12) and melanoma (*n* = 16) samples. Moreover, we obtained TCGA-skin cutaneous melanoma (TCGA-SKCM) patient mRNA expression matrix (*n* = 458) and corresponding clinical data from UCSC-Xena (http://xena.ucsc.edu/) (21) for expression analysis, gene set enrichment analysis, survival analysis, staging analysis, TME analysis, and correlation analysis.

### Construction of Weighted Co-expression Network in Melanoma Patients

WGCNA aims to mine functionally related genes with similar co-expression patterns (22). Expression correlation coefficients were applied to measure intergenic co-expression relationships, and genes with high correlation coefficients were assigned to the same module and presented similar expression patterns. These highly correlated module genes may be involved in the same biological process or pathway. Gene co-expression networks were constructed using the WGCNA package in R (4.1.1) by the top 25% of the median absolute deviation (MAD) ranked genes in the GSE91061 expression matrix normalized by log2(count+1). The co-expression similarity matrix for all genes was generated using the average linkage method and subsequently converted into an adjacency matrix to ensure the construction of an unsigned scale-free network. The weighted adjacency matrix was further transformed into a topological overlap matrix (TOM) to estimate the connectivity of the unsigned network. A hierarchical clustering approach was adopted to construct the clustering tree structure of the TOM, and dynamic hybrid cuts were achieved by the variability of the TOM with a cut height of 0.25. Gene modules were fused under the variability of the estimated module eigengenes (MEs) and represented by different colors.

### Identification of the Co-expression Gene Module Associated With CAFs

First, 56 marker genes (Supplementary Table 1), associated with CAFs and reported in many pan-cancer CAFs studies, were collected from Mao et al.'s review (14). Then, according to the marker genes, the CAFs score was calculated for each patient in GSE91061 (*n* = 49) using the GSVA algorithm. Finally, the correlation between the CAFs score and MEs was assessed using Pearson's correlation coefficient, and the gene module with the smallest *P-*value was selected as the most relevant module for the CAFs score. Genes in the module were defined as highly correlated with CAFs.

### Functional Enrichment Analysis of Module Genes

For the biological functions of the module genes associated with CAFs, enrichment analyses of Gene Ontology (GO) (23), Kyoto Encyclopedia of Genes and Genomes (KEGG) (24), Reactome (REAC) (25), and WikiPathways (WP) (26) were executed in g:Profiler online tool (https://biit.cs.ut.ee/gprofiler/gost) (27). Entries with *FDR* < 0.05 were considered significant and visualized by Hiplot (https://hiplot.com.cn).

### Identification of Key Genes by LASSO Regression Analysis and Random Forest Algorithm

RNA-seq count data from GSE91061 were normalized with TMM (trimmed mean of M values) and analyzed using the edgeR package (28), and genes with *P* < 0.05 were defined as anti-PD-1 response differentially expressed genes (DEGs). The intersection between module genes associated with CAFs and DEGs related to anti-PD-1 therapy response were extracted and defined as CAFs-related DEGs (CDEGs), and further LASSO and random forest analysis were adopted to identify key CDEGs (KCDEGs) using the glmnet package (nlambda = 1000, 10-fold cross-validation) and randomForest package. LASSO regression was characterized by variable selection and regularization while fitting a generalized linear model, which aids it well-adapted to the linear and non-linear operations (29) and random forest is a popular classifier that can handle input samples without dimensionality reduction to generate an unbiased estimate of the error in the process of forest building (30).

### Gene Set Enrichment Analysis for KCDEGs

Once 458 TCGA-SKCM samples were divided into two groups with the median gene expression, the edgeR package was further employed for differentially expressed genes (*FDR* < 0.05) between the two groups. Subsequently, based on hallmark gene sets of the MSigDB (http://www.gsea-msigdb.org/gsea/msigdb/index.jsp) (31), GSEA was utilized (*FDR* < 0.05) to estimate the function of KCDEGs by clusterProfiler package (32).

### Survival and Staging Analysis of KCDEGs in Melanoma Patients

To investigate whether KCDEGs have an impact on survival and disease progression in melanoma patients, we applied gene expression analysis (One-way ANOVA, *P* < 0.05), survival analysis (Log-rank *P* < 0.05), and staging analysis [One-way ANOVA, *Pr(*>*F)* < 0.05] in TCGA-SKCM patients by GEPIA (33). In addition, we evaluated the predictive effect of combined KCDEGs on patients' prognosis in the TCGA-SKCM cohort (*n* = 458). A multivariate Cox proportional risk regression model was constructed for the KCDEGs using the survival R package (34), as well as regression coefficients were retained for each KCDEG. And risk score was calculated for each patient according to the following equation.


$$\begin{array}{l} \text{Risk score}=\underset{i}{\sum }Coefficient{\left(KCDEG_{i}\right)}^{*}Expression\left(KCDEG_{i}\right) \end{array}$$

SKCM patients were assigned to low- and high-risk groups based on the median risk score calculated above. We finally assessed the overall survival between the two risk groups by the Kaplan-Meier curve.

### Association Assessment of KCDEGs With TME Signature

The RNA-seq count matrix of TCGA-SKCM was converted into TPM (transcripts per million) values for the next analysis. The variation of TME for each sample and 64 xCell TME signature were investigated by the xCell online tool (https://xcell.ucsf.edu/) (35) to estimate the infiltration level of TME-associated cells (mainly include immune cells and stromal cells). Pearson correlation analysis was utilized to calculate the degree of correlation between KCDEGs and the infiltration levels of TME-associated cells (*P* < 0.05). We used the Wilcoxon test to compare the TME-associated cell infiltration level between low- and high-risk groups (*P* < 0.05).

### Correlation Analysis of CAFs-Related Stimulators and Immunotherapy-Related Genes

The mRNA expression data for KCDEGs, CAFs-related stimulators, antitumor immune-related genes and, immune checkpoint-related genes were extracted from the TPM matrix of TCGA-SKCM. Spearman correlation analysis was adopted to calculate the correlation coefficient between each gene (*P* < 0.05). And the ggcorrplot R package (36) was employed for the correlation coefficient visualization.

### Diagnostic Performance Evaluation and Validation of KCDEGs

To further assess the predictive ability of KCDEGs for anti-PD-1 treatment response, the pROC package (37) was chosen to calculate the area under the curve (AUC) of the receiver operating characteristic (ROC) curve for each KCDEGs in discovery and validation sets. Higher AUC values represent a better gene diagnostic performance. By binary logistic regression (SPSS, v25.0), KCDEGs were firstly fitted to the CAF-derived gene panel, and the diagnostic performance of the CAF-derived gene panel was assessed through ROC curves.

To determine the single-cell expression distribution of these KCDEGs, we analyzed the expression of each KCDEGs with single-cell RNA-seq data by Tumor Immune Single Cell Hub (TISCH) (38). Six SKCM datasets were included in the analysis: four non-treatment cohorts (GSE123139, GSE139249, GSE148190 and GSE72056), one anti-PD-1 treatment cohort (GSE115978) and one anti-PD-1/anti-CTLA-4 treatment cohort (GSE120575).

### Statistical Methods

Statistical analyses and visualization were performed with R software (version 4.1.1). Normally distributed continuous variables between two groups were compared by *t*-test. Otherwise, the Wilcoxon test was applied. The *P*-values for GSEA were corrected by the Benjamini-Hochberg method. The statistical significance of survival analysis was assessed by the Log-rank test and corrected by the Bonferroni method. Correlations between variables were explored by Pearson or Spearman coefficients. All statistical tests were two-sided.

## Results

### Construction of Weighted Gene Co-expression Networks and Identification of CAFs-Related Modules

**Figure 1:**
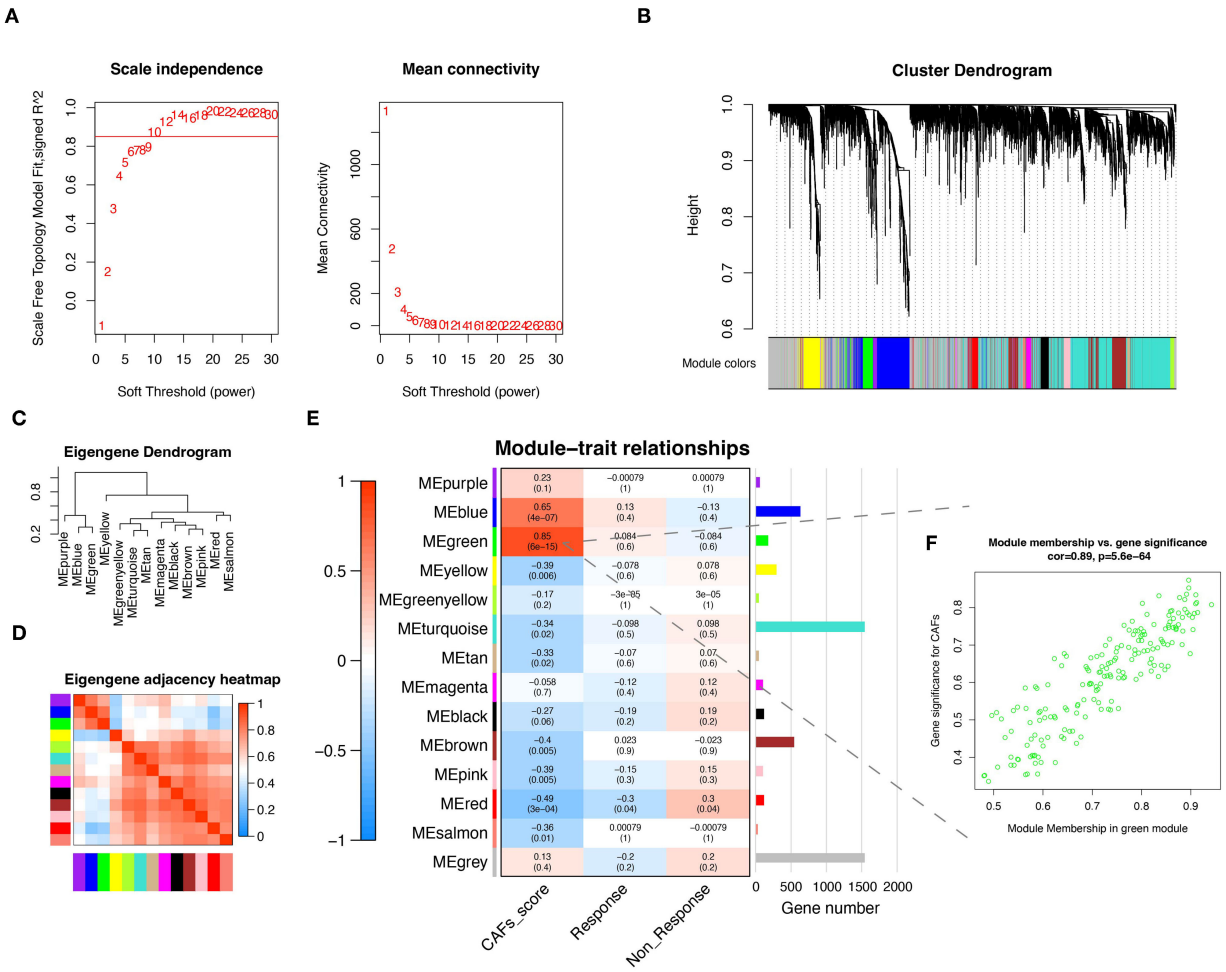
CAFs related gene module was identified by WGCNA. **(A)** The test of scale independence and mean connectivity for constructing scale-free network. **(B)** Hierarchical clustering dendrogram of co-expressed genes after module fusion. **(C)** Eigengene dendrogram of function module. **(D)** Eigengene adjacency correlation heatmap of the function module. **(E)** Heatmap of the correlation between module and trait. **(F)** Correlation scatter plot of gene significance for CAFs and module membership from green module. WGCNA, Weighted gene co-expression network analysis.

To obtain the gene modules associated with CAFs, we constructed a weighted gene co-expression network by WGCNA for GSE91061. A soft threshold of β = 10 (Figure 1A) was chosen for the scale-free topological network model fitting (R2 = 0.85). Subsequently, 14 gene modules were obtained by average linkage hierarchical clustering, TOM dynamic hybrid cut (cutHeight = 0.25), and module fusion, with gray as a non-functional module and the rest as functional modules (Figure 1B). Figures 1C,D demonstrated the similarities and differences among these modules. Similar module genes may be involved in similar biological function regulation. Finally, the Pearson correlation analysis was performed to calculate the correlation of MEs with the CAFs score and clinical characteristics (Non-response and Response). The results suggest that the green module was the most relevant to the CAFs score, which included 184 module genes (Figure 1E). Further, we displayed a significant correlation between module membership in the green module and gene significance for CAFs (Figure 1F, Cor = 0.89, *P* = 5.6E-64).

### Biological Functions of Genes in the Green Module

**Figure 2:**
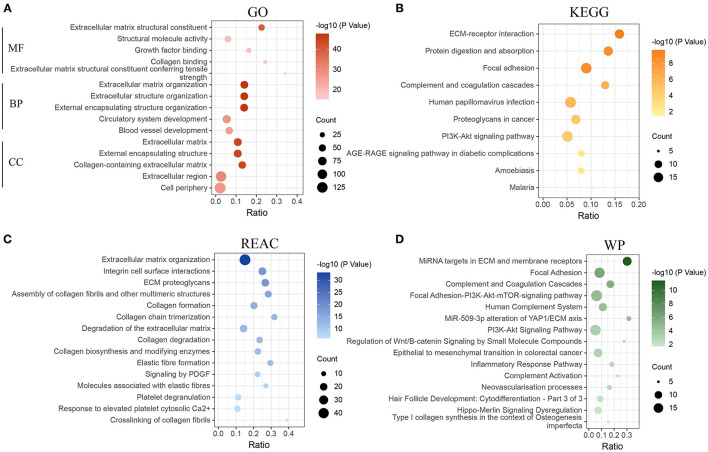
Functional enrichment analysis of genes in the green module. **(A)** Enrichment analysis of gene ontology (GO). **(B)** Enrichment analysis of Kyoto Encyclopedia of Genes and Genomes pathways (KEGG). **(C)** Enrichment analysis of Reactome pathways (REAC). **(D)** Enrichment analysis of WikiPathways (WP). MF, molecular function; BP, biological process; CC, cellular component.

The green module was identified as the most associated gene module with CAFs. To further explore the functional association between green module genes and CAFs, enrichment analysis (*FDR* < 0.05) of module genes was realized based on GO, KEGG, REAC, and WP. GO results exhibited that the molecular function of 184 genes is mainly enriched in extracellular matrix structural constituent, structural molecule activity, and growth factor binding (Figure 2A). The involved terms of biological processes and cellular components include extracellular matrix organization, extracellular structure organization, extracellular matrix, and collagen-containing extracellular matrix. The above results demonstrated the significant association of module genes with ECM remodeling. Increasing evidence has revealed that CAFs are major contributors to ECM remodeling in the TME (14, 39, 40). CAFs can alter the structure, arrangement, and stiffness of the ECM by secreting various matrix proteins, growth factors, and cytokines, which in turn affect the migration and invasion of cancer cells (41). Another study emphasized that CAFs can also promote the expression of fibronectin and laminin through the secretion of cytokine TGF-β1, which affects the remodeling process of ECM (42). Subsequent pathway enrichment results of KEGG, REAC, and WP similarly demonstrated the close association of these module genes with ECM (Figures 2B–D), such as ECM-receptor interaction, ECM proteoglycans, and miRNA targets in ECM, and membrane receptors. Notably, the enrichment results for KEGG and WP presented significant associations of these genes with the PI3K-AKT signaling pathway (KEGG: *FDR* = 2.4E-05; WP: *FDR* = 4.9E-04), suggesting that CAFs-related genes may be involved in the regulation of the PI3K-AKT signaling pathway. The previous study has revealed that CAFs can regulate proliferation, apoptosis and, invasion of lung cancer cells by activating the PI3K-AKT-mTOR signaling pathway through secretion of IL-22 (43).

### Identification of Key CAFs-Related Differential Genes by Machine Learning

**Figure 3:**
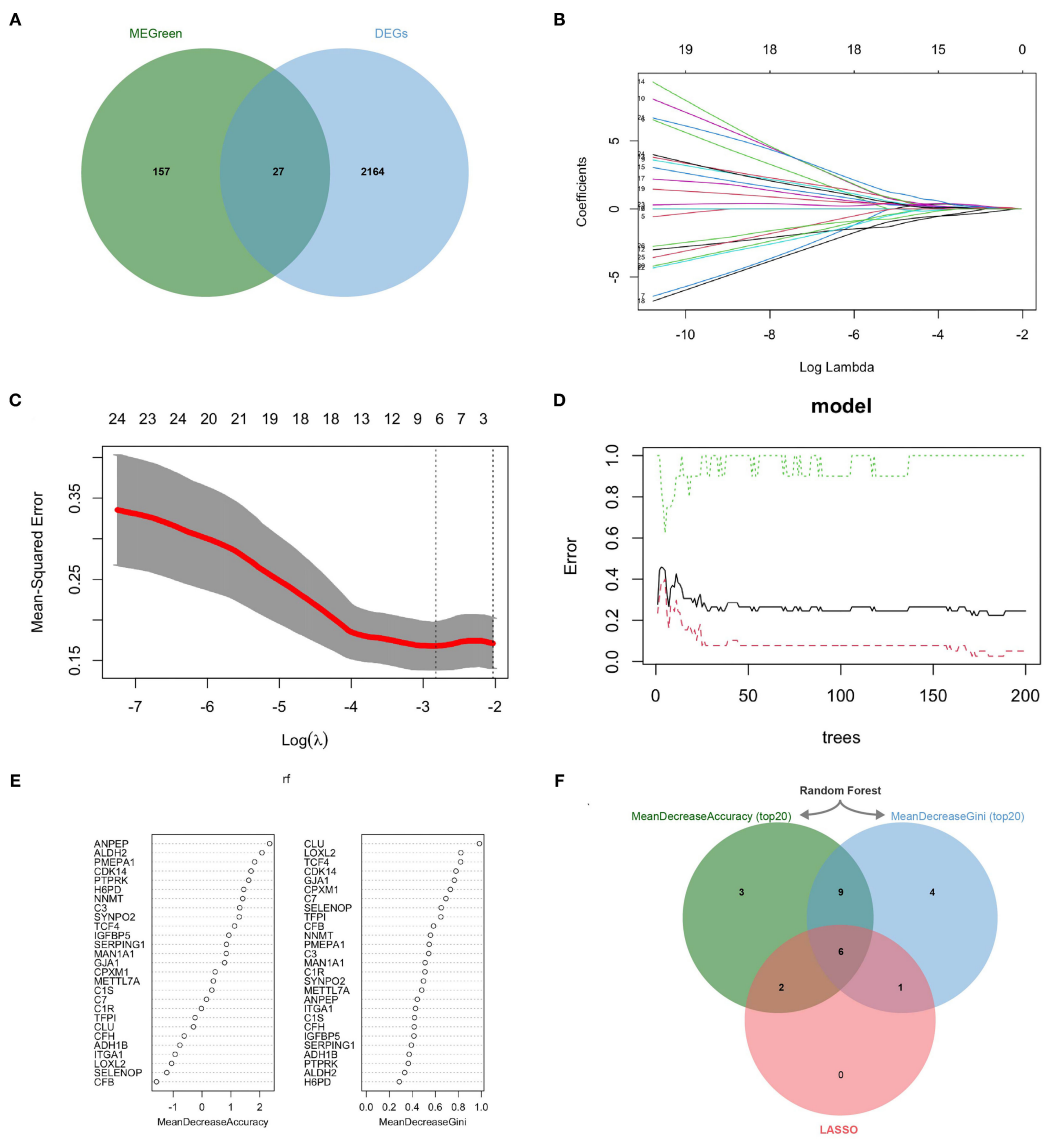
KCDEGs were identified by the LASSO and random forest algorithm. **(A)** Venn diagram showing the intersection between the green module genes and the differential anti-PD-1 treatment response genes. **(B,C)** LASSO was used to determine the optimal gene variables, and the model achieved the best performance when log(λ) was set to −2.788. **(D,E)** Random forest was used to screen gene variables, 150 trees were selected to build a robust model, and genes were sorted according to mean decrease accuracy and mean decrease Gini. **(F)** Venn diagram of six KCDEGs identified by two machine learning algorithms. KCDEGs, key CAFs-related differentially expressed genes; LASSO, least absolute shrinkage and selection operator.

2191 DEGs were retained by performing gene differential analysis of anti-PD-1 treatment-responsive and non-responsive patients in the GSE91061 dataset. Subsequently, we obtained 27 CDEGs with the intersection of 184 CAFs-related genes and 2191 DEGs (Figure 3A). To further identify the KCDEGs, a linear model was first constructed for the 27 CDEGs using LASSO regression with variable screening and regularization (Figure 3B). The mean-squared error of the model was minimized when log(λ) = −2.788 (Figure 3C). Finally, we screened nine genes as the best variables to distinguish patients responding and non-responding to anti-PD-1 treatment. Concurrently, a random forest classifier was built based on 27 CDEGs, and we selected ntree = 150 to ensure the stability of the constructed model (Figure 3D). Then all molecules were ranked by mean decrease accuracy and mean decrease Gini (Figure 3E). The intersection of the top20 genes ranked by two methods in the random forest model and the 9 genes identified by LASSO was extracted. Finally, we obtained 6 KCDEGs identified by two machine learning algorithms (Figure 3F).

### Expression Analysis of KCDEGs

KCDEGs may display diversified expression patterns in different patients, and we further investigated their expression between patients responding and non-responding to anti-PD-1 therapy. Supplementary Figure 2A showed that *CDK14* (Cyclin Dependent Kinase 14), *SYNPO2* (Synaptopodin 2), *TCF4* (Transcription Factor 4), and *TFPI* (Tissue Factor Pathway Inhibitor) expression were significantly elevated in the response group (Student's *t*-test, *P* < 0.05). Although the expression of *GJA1* (Gap Junction Protein Alpha 1) and *CPXM1* (Carboxypeptidase X, M14 Family Member 1) had no significant change, *CPXM1* tended to increase somewhat in the non-response group (Student's *t*-test, *P* = 0.054). In addition, we investigated the expression of KCDEGs in GSE114445 (Supplementary Figure 2B). Among them, expressions of *CDK14, SYNPO2, TCF4*, and *GJA1* displayed a gradual decrease in normal skin, nevus and melanoma samples. In contrast, the expression of *TFPI* was significantly increased in melanoma samples compared to nevus (Student's *t*-test, *P* < 0.05), but without change in normal skin (Student's *t*-test, *P* = 0.73). Notably, *CPXM1* expression demonstrated a progressive or gradual increasing trend in normal skin, nevus, and melanoma samples while decreasing in patients responding to anti-PD-1 therapy, which showed the gene might serve as a pro-cancer molecule in melanoma.

### Gene Set Enrichment Analysis of KCDEGs

**Figure 4:**
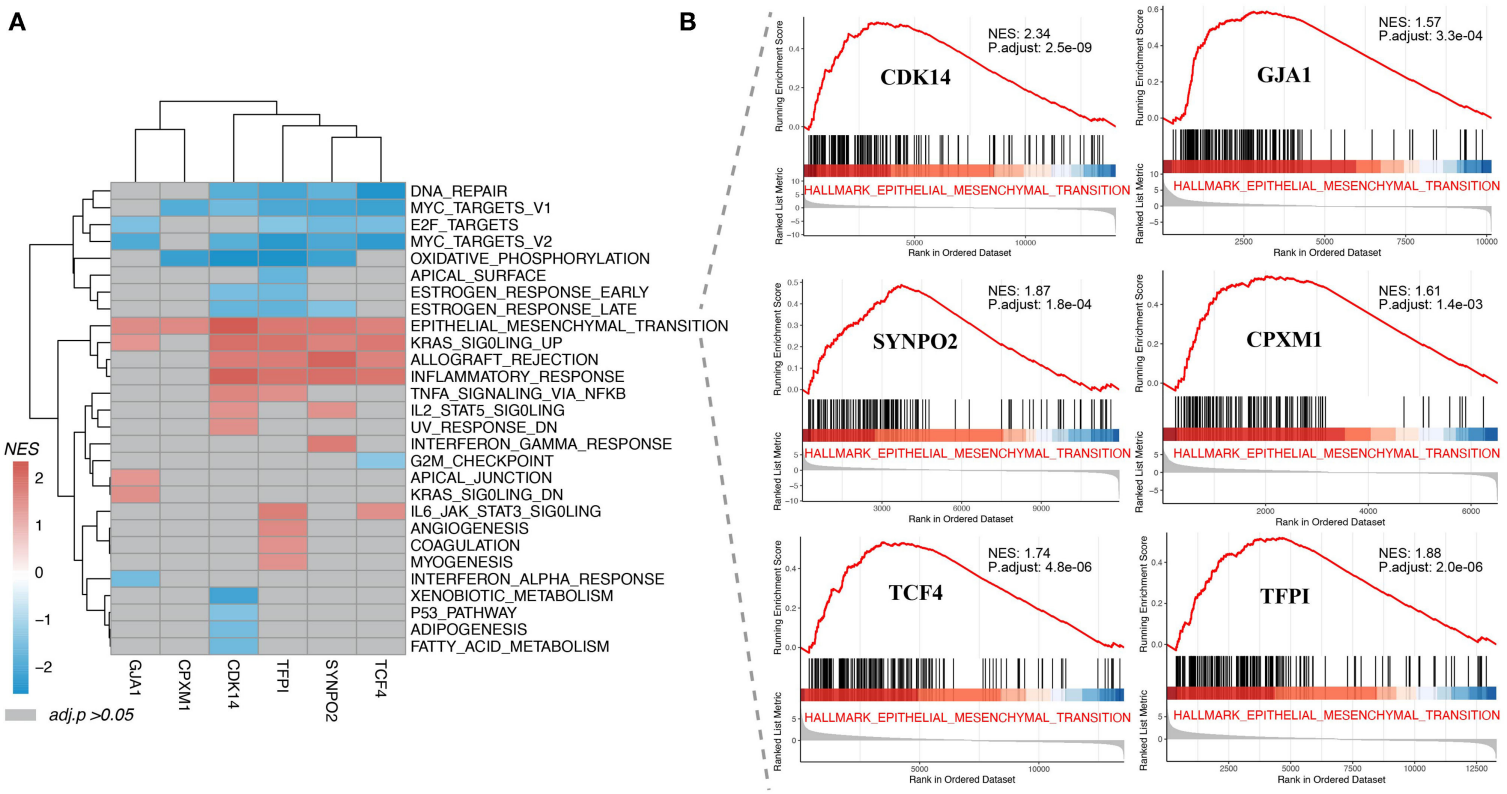
Gene set enrichment analysis of KCDEGs. **(A)** Heatmap depicting 28 cancer hallmarks enriched by KCDEGs. Red or blue means *FDR* < 0.05 and gray means *FDR* > 0.05. **(B)** All KCDEGs were enriched in the epithelial-mesenchymal transformation pathway.

As shown in Figure 4A, all enriched KCDEGs in 28 cancer hallmarks (*FDR* < 0.05) were involved in their respective biological processes in a certain state. For example, the enriched KCDEGs-related genes involved in DNA repair, MYC targets, E2F targets, and oxidative phosphorylation were repressed, while others were activated in epithelial-mesenchymal transition (EMT), KRAS signaling up, allograft rejection, and inflammatory response. Moreover, EMT was the most significant activated term and closely associated with all KCDEGs (*CDK14*: *NES* = 2.34, *FDR* = 2.5E−09; *GJA1*: *NES* = 1.57, *FDR* = 3.3E−04; *SYNPO2*: *NES* = 1.87, *FDR* = 1.8E−04; *CPXM1*: *NES* = 1.61, *FDR* = 1.4E−03; *TCF4*: *NES* = 1.74, *FDR* = 4.8E−06; *TFPI*: *NES* = 1.88, *FDR* = 2.0E−06) (Figure 4B). Taken together, the above results fully demonstrate the close association of KCDEGs with cancer development, especially the regulatory association with EMT confirmed in several studies (44–46). In addition, previous studies have identified an association between EMT and elevated PD-1 levels in lung adenocarcinoma patients (47). This evidence suggests a potential link among CAFs, EMT, and anti-PD-1 therapy response.

### Survival Impact and Staging Expression of KCDEGs on Melanoma Patients

**Figure 5:**
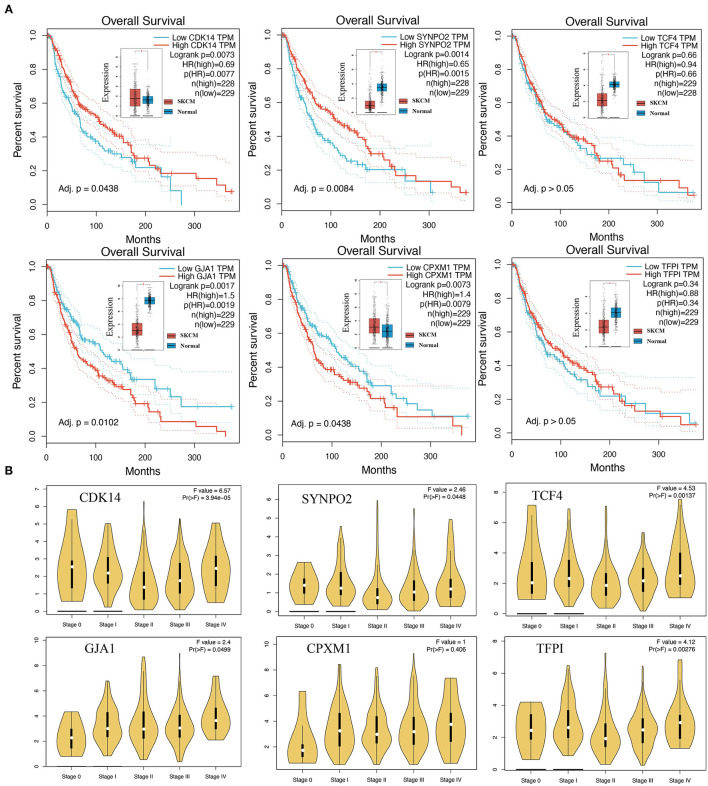
The association of KCDEGs with survival and staging was determined by GEPIA. **(A)** Kaplan-Meier curve showing the survival of patients with high and low expression of KCDEGs based on TCGA-SKCM. The built-in box plot shows the expression level of KCDEGs in SKCM and normal samples. **(B)** Violin diagram showing the expression level of KCDEGs in samples with different TCGA-SKCM stages. SKCM, skin cutaneous melanoma.

Based on mRNA expression and clinical data of TCGA-SKCM, Kaplan-Meier curves demonstrated that high expression of *CDK14* (Log-rank *P* = 0.0073, *HR* = 0.69) and *SYNPO2* (Log-rank *P* = 0.0014, *HR* = 0.65) is tended to predict better overall survival (OS) (Figure 5A). In contrast, high expression of *GJA1* (Log-rank *P* = 0.0017, *HR* = 1.50) and *CPXM1* (Log-rank *P* = 0.0073, *HR* = 1.40) was associated with worse OS in patients. To further examine the association between KCDEGs and tumor stage, the mRNA expression distribution of KCDEGs in patients with different tumor stage was investigated, and we can observe a trend of elevated expression of *GJA1* [*F* = 2.4, *Pr(*>*F)* = 0.0499] and *CPXM1* [*F* = 1.0, *Pr(*>*F)* = 0.406] with advanced stage, although *CPXM1* was not significant (Figure 5B).

### The Role of KCDEGs in the Melanoma TME

**Figure 6:**
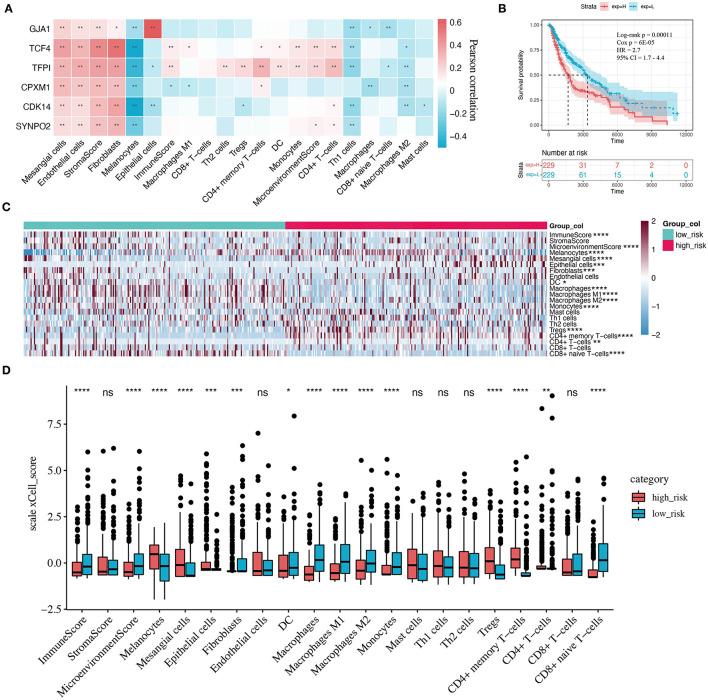
Correlation between KCDEGs and tumor microenvironment (TME) components. **(A)** Heatmap of the Pearson correlation between KCDEGs and 21 TME features. *, *P* < 0.05; **, *P* < 0.01. **(B)** Kaplan-Meier curve showing the survival difference of patients between high and low risk score groups. **(C,D)** Heatmap and box plot depicting differences of TME features in two risk score groups. Wilcoxon test, *, *P* < 0.05; **, *P* < 0.01; ***, *P* < 0.001; ****, *P* < 0.0001.

TME was suggested to have an important effect on the progression of malignant tumors and response to anti-PD-1 therapy (48, 49), which is confirmed by our findings. Twenty-one TME signature fractions significantly correlated with KCDEGs were traced in our further investigation (Pearson correlation, *P* < 0.05). As demonstrated in Figure 6A, KCDEGs were significantly and positively correlated with stromal fractions such as fibroblasts, endothelial cells, and mesangial cells, but a significant negative correlation with melanocytes. In the analysis of the relationship between KCDEGs and immune fractions, *TCF4* and *TFPI* were positively associated with immune score (*P* < 0.01), while *CPXM1* showed a negative association (*P* < 0.05). Meanwhile, *TCF4* and *TFPI* were positively related to CD4^+^ memory T cells, dendritic cells (DCs), monocytes, CD4^+^ T cells (*P*< *0*.05) and negatively related to Th1 and macrophages M2 (*P* < 0.05). *CPXM1* was only positively correlated with CD4^+^ memory T cells (*P* < 0.05) and negatively correlated with macrophages (*P* < 0.05).

The complexity of the relationship between KCDEGs and TME signature also indicates that they act as different mechanisms in TME. To better evaluate the ability of combined KCDEGs impacting on survival and TME, we performed multivariate Cox regression analysis and calculated risk score according to the following equation:


$$\begin{array}{l} \text{Risk score}=-0.132^{*}Exp_{CDK14}-0.088^{*}Exp_{SYNPO2}\\ \;-0.114*Exp_{TCF4}+0.157^{*}Exp_{GJA1}\\ \;+0.095^{*}Exp_{CPXM1}+0.072^{*}Exp_{TFPI} \end{array}$$

Kaplan-Meier curve demonstrates that the high risk score group (high-risk group) has worse survival (Log-rank *P* = 0.00011, Cox *P* = 6E−05, *HR* = 2.7, 95% *CI* 1.7–4.4) (Figure 6B). In addition, there is a significant difference in TME between the high- and low-risk groups (Figures 6C,D). In particular, compared with the low-risk group, the components of the immune score and macrophages, monocytes, as well as CD8^+^ naïve T cells are lower (Wilcoxon test, *P* < 0.0001), while the components of melanocytes, Tregs, and CD4^+^ memory T cells are higher in the high-risk group (Wilcoxon test, *P* < 0.0001).

### Association of KCDEGs With CAFs-Related Stimulators and Immunotherapy-Related Molecules

Correlation analysis was applied in six KCDEGs (*SYNPO2, CDK14, CPXM1, TFPI, TCF4*, and *GJA1*), five CAFs-related stimulating factors (*LIF, HSF1, IL6, IL1B*, and *CXCL12*), five antitumor immune-related molecules (*CXCL10, CXCL9, CXCR3, CCL5*, and *CCL4*), and five immune checkpoint-related molecules (*TIGIT, LAG3, CTLA4, CD274*, and *PDCD1*). KCDEGs displays significant positive correlations (Spearman correlation, *P* < 0.05) with most of the molecules (Supplementary Figure 3), which implies KCDEGs and the highly correlated molecules may perform similar functions and play a synergistic regulatory role in tumor development (50). But *CPXM1* and *GJA1* did not show significant correlations with antitumor immune-related molecules and immune checkpoint-related molecules. Notably, *HSF1* was negatively correlated with all other molecules, suggesting that it may exert the opposite regulatory function to them.

We can conclude the following: (i) KCDEGs may affect the secretion of CAFs-related stimulating factors or participate in the activation of CAFs. For example, IL-1β and IL-6 are pro-inflammatory cytokines that can promote the activation of CAFs through NF-κB and JAK-ROCK-STAT3 signaling pathways, thus causing tumor progression (51, 52). Heat shock factor 1 (HSF1) regulates the pro-tumor effects of CAFs by activating β-catenin and YAP/TAZ signaling pathways (53). (ii) Antitumor immune-related molecules including CXCL10, CXCL9, CXCR3, CCL5, and CCL4 belong to the chemokine family and have been reported in several studies to recruit immune cells such as cytotoxic T lymphocytes (CTL) and NK cells to kill tumor cells (54). KCDEGs may generate similar tumor-suppressive effects with the five molecules above. (iii) The high positive correlation of KCDEGs with immune checkpoint-related molecules implies that they may affect the benefit from ICB therapy and turn into potential targets for immunotherapy.

### Evaluation and Validation of Response Prediction Performance of KCDEGs for Anti-PD-1 Treatment

**Figure 7:**
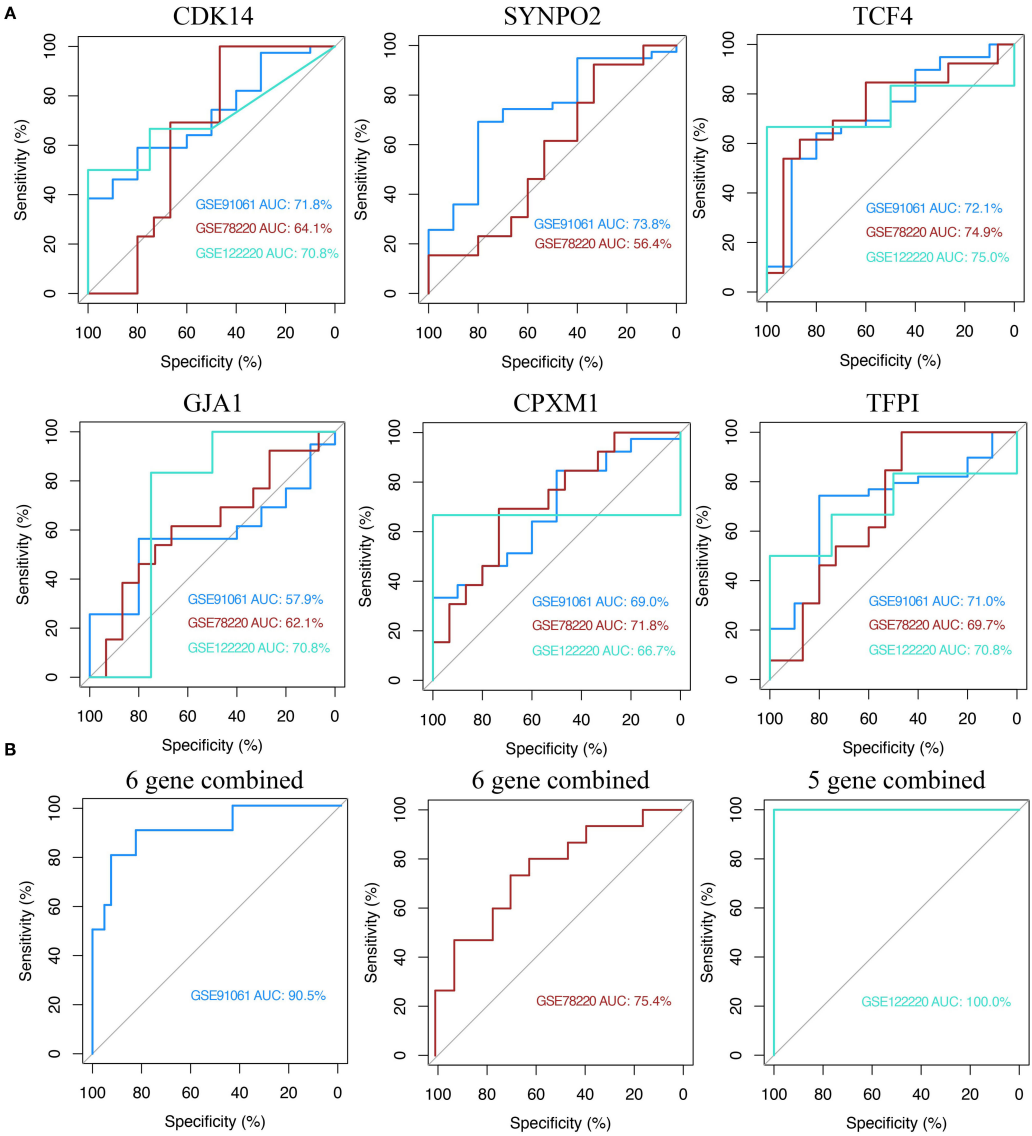
Receiver operating characteristic (ROC) curve analysis of KCDEGs of discovery set and two validation sets. **(A)** Independent diagnostic performance of KCDEGs in discovery set (GSE91061) and two validation sets (GSE78220 and GSE122220). No corresponding probe of SYNPO2 was found in GSE122220. **(B)** Combined diagnostic performance of KCDEGs in the discovery set and two validation sets. AUC, area under the curve.

The above results of the correlation analysis have suggested a strong association between KCDEGs and ICB therapy. Six KCDEGs (except SYNPO2, whose corresponding probe was not found in GSE122220) were evaluated in discovery and validation sets to predict the response to anti-PD-1 therapy by ROC curves. The predictive power of the ROC was inferred by calculating its AUC, with a larger AUC indicating better prediction. In the discovery set, *CDK14, SYNPO2, TCF4* and *TFPI* are the best predictors (AUC > 70%) (Figure 7A). In the two validation sets, the AUC ranges from 56.4 (*SYNPO2* in GSE78220) to 75% (*TCF4* in GSE12220). Overall, *TCF4* possessed the most stable prediction effect (GSE91061: AUC = 72.1%; GSE78220: AUC = 74.9%; GSE122220: AUC = 75.0%) and may be a promising anti-PD-1 treatment response predictor. Concurrently, combined KCDEGs, have a satisfactory prediction effect beyond the single evaluation, where there is 90.5% AUC in the discovery sets, while 75.4 and 100% AUC in the two validation sets, respectively (Figure 7B).

### Summary of Features and the Single-Cell Localization of KCDEGs

**Figure 8:**
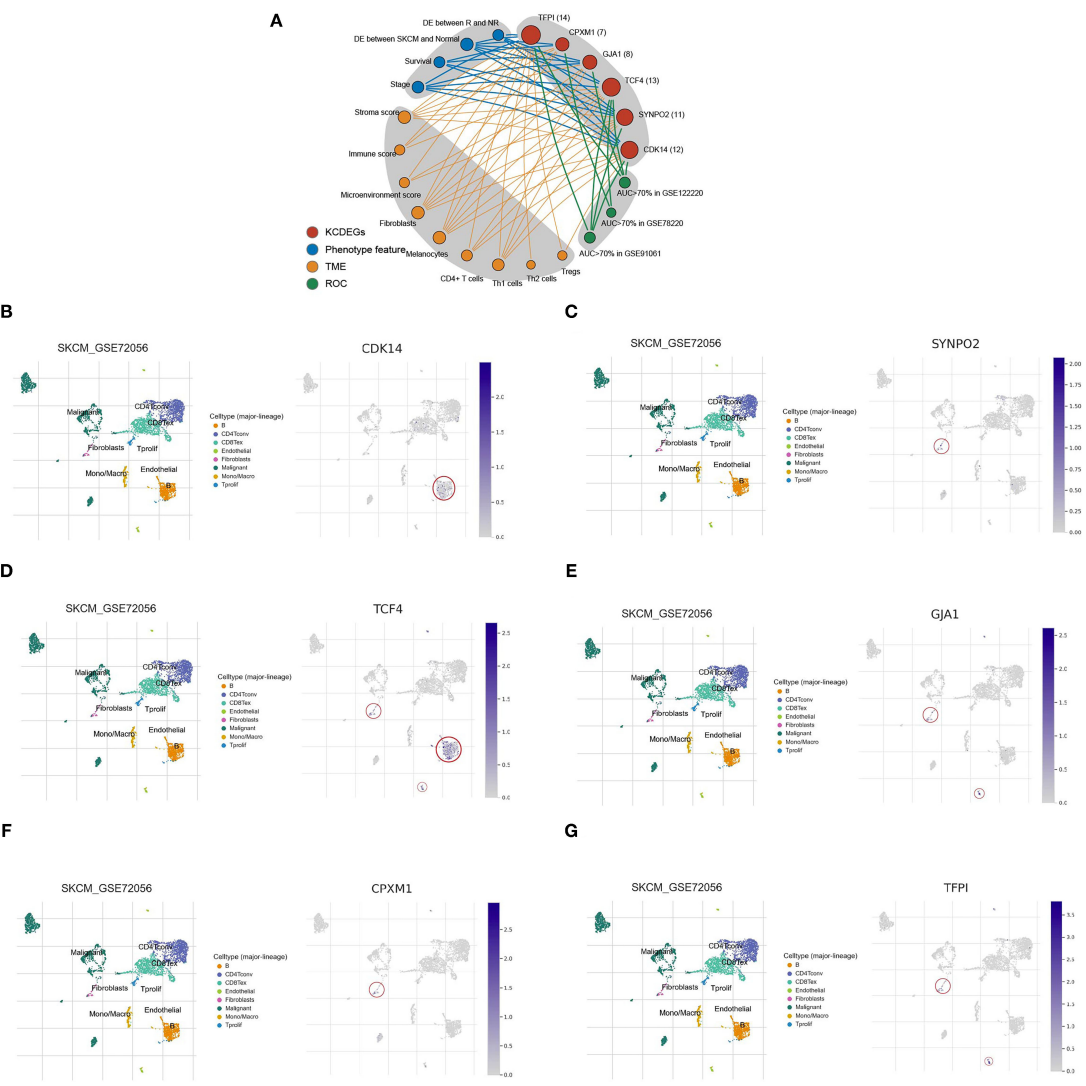
Characteristic network and single-cell analysis of KCDEGs. **(A)** Multidimensional feature network of KCDEGs. Different feature nodes are marked by different colors, and a larger circle means more nodes are connected. **(B–G)** Heatmap showing KCDEGs expression of different cell types in six single-cell RNA-seq datasets analyzed by Tumor Immune Single Cell Hub, and the built-in single-cell UMAP map depicting the expression distribution of KCDEGs in SKCM single-cell dataset GSE72056. DE, different expression.

The above multidimensional systematic analysis aided in better dissecting the features of KCDEGs and their impacts of TME. To intuitively determine the importance of each KCDEG, a multi-feature linkage network of KCDEGs was constructed, which includes KCDEGs, phenotypic features (Stage, Survival, DE between SKCM and Normal, DE between R and NR), TME (Stroma/Immune/Microenvironment score, Fibroblasts, Melanocytes, CD4^+^T/Th1/Th2/Tregs cells) and ROC (Figure 8A). Each KCDEGs has respective nodes: *TFPI* (nodes = 14), *TCF4* (nodes = 13), *CDK14* (nodes = 12), *SYNPO2* (nodes = 11), *GJA1* (nodes = 8), and *CPXM1* (nodes = 7), of which more nodes in the network are more important.

The results of the single-cell analysis demonstrated that *CDK14* is predominantly distributed in B cells (Figure 8B), *SYNPO2* presented highest expression in fibroblasts (Figure 8C), *TCF4* mainly enriched in fibroblasts, B cells, and endothelial cells (Figure 8D), *GJA1* presented highest expression in fibroblasts and endothelial cells (Figure 8E), *CPXM1* mainly distributed in fibroblasts (Figure 8F) while *TFPI* exhibited the highest expression in fibroblasts and endothelial cells (Figure 8G). Additionally, the results were verified in the other five datasets (Supplementary Figure 4).

## Discussion

Although anti-PD-1 therapy is expensive and exerts adverse effects, anti-PD-1 immune checkpoint inhibitors have been confirmed of excellent clinical activity in melanoma treatment (55). However, not all patients can produce an objective response to it (4). It is essential and urgent to determine the patients' response using reliable biomarkers to predict anti-PD-1 efficacy before the treatment. CAFs can suppress antitumor immune responses through upregulation of PD-1 expression, and CAFs-related genes may be the potential markers in predicting the efficacy of anti-PD-1 immune checkpoint inhibitors (56–58). In this study, we successfully elucidated the potential regulatory efficacy of CAFs-related genes in TME and discovered a six-gene panel for prediction of response to anti-PD-1 therapy. Our discovery manifests the panel is favorably consistent with the clinical response of patients, which can aid in the better clinical management of melanoma patients.

We first calculated the CAFs score for each patient based on 56 CAFs markers reported in literature through the GSVA algorithm. Subsequently, WGCNA was applied to construct gene co-expression networks, which has the potential to identify CAFs-related genes (59),and the green module from networks was of the highest correlation with the CAFs score. By intersecting green module genes and the DEGs, we obtained 27 response-associated CDEGs for anti-PD-1 therapy. Finally, 6 KCDEGs (*CDK14, SYNPO2, TCF4, GJA1, CPXM1*, and *TFPI*) were identified by the methods of lasso regression and random forest.

Comprehensive analysis implied that KCDEGs are closely associated with CAFs and appeared as potential predictors of anti-PD-1 therapy efficacy in melanoma. First, gene expression analysis demonstrated six KCDEGs expressed with significant differences could reflect patients' response status to anti-PD-1 therapy, with *CDK14, SYNPO2, TCF4, TFPI* up-regulated in responders. In addition, the expression of six KCDEGs presented significant differences in SKCM. Secondly, GSEA indicated that KCDEGs were enriched in several cancer hallmarks such as EMT, MYC targets, KRAS signaling, and inflammatory response. EMT is the process epithelial cells acquire a mesenchymal phenotype after downregulating epithelial features, which exhibited crucial functions in cancer progression. Previous studies confirmed that EMT could promote CAF activation and alter the secretory phenotype of CAFs in the mesenchyme (60–62). Additionally, *MYC* is one of the widely studied oncogenes, and high *MYC* targets scores in breast cancer were associated with increased tumor immune cell infiltration (63). Moreover, CAFs have been verified to have complex crosstalk in KRAS-driven tumorigenesis and to influence immune regulation through the immune checkpoint (64). Taken together, KCDEGs may be a vital part of the melanoma immune infiltration regulatory network and suggest a predictive effect on the response to anti-PD-1 therapy.

Survival analysis shows that high expression of *CDK14* and *SYNPO2*, plus low expression of *GJA1* and *CPXM1* were associated with better survival in SKCM patients. As patients with excellent prognoses often displayed positive responses to anti-PD-1 therapy (65), we speculated that the combination of *CDK14* and *SYNPO2* (high expression), as well as *GJA1* and *CPXM1* (low expression), may be associated with a positive response to anti-PD-1 therapy. As an activator of the WNT pathway through mediating the phosphorylation of LRP6 in the G2/M phase, *CDK14* was found that it could promote the progression of several cancers (66–68). However, our findings suggest that high expression of *CDK14* is associated with better prognosis and anti-PD-1 therapy response in melanoma patients, implying a unique mechanism of *CDK14* in melanoma. As expected, *SYNPO2*, whose promotor methylation and low transcriptional expression were found to associate with metastasis and poor clinical outcome in melanoma (69), can be a potential predictor to provide new therapeutic strategies in this study. *GJA1* is a gene encoding the protein connexin 43 (Cx43) that is a vital component of gap junctions and was disclosed to be of an important role in communication between tumor cells and surrounding immune cells like NK and dendritic cells (DC) (70). The up-regulation of *GJA1* expression implicates a crucial link between melanoma cells and endothelial cells, thus enhancing tumor metastasis in melanoma (71). Interestingly, Cx43 has generally been reported as a potential cancer suppressor to improve therapeutic efficacy against melanoma (72). *CPXM1* is suggested as an immune-related gene to predict cancer prognosis (73, 74), which is consistent with our findings.

Correlation analysis of KCDEGs with various fractions in TME revealed all these six KCDEGs (*TFPI, TCF4, CDK14, SYNPO2, GJA1*, and *CPXM1*) positively correlated with stroma score, endothelial cells, and fibroblasts (Figure 6A), which can be promisingly accountable for tumor progress and prognosis of melanoma patients. For example, *TFPI*, encoding tissue factor pathway inhibitor, is closely associated with the endothelial cell surface and can regulate angiogenesis (75). Notably, endothelial cell activation requires the elevated expression of Snail1, which can strengthen CAF activation in a paracrine manner promoting immune infiltration in TME by secreting IL-6 in tumor development (9, 76). All of these are witnessed by the co-culture of CAFs with endothelial cells under hypoxic conditions promoting breast cancer angiogenesis (77) and tumor exposed-lymphatic endothelial cells (teLEC) reported as the promoter of cancer cell invasion and tumor cell proliferation by regulating IL-6 (78). Taken together, we believe TFPI may mediate the action of CAFs and endothelial cells through transcription factors and cytokines, thereby regulating immune cell infiltration in TME, which is also investigated from the correlation analysis of KCDEGs-immune cell fractions. *TCF4* and *TFPI*, up-regulated in the PD-1 therapy response group and low expressed in the SKCM group (Figures 5A,B), were positively related to CD4+ memory T cells, CD4+ T cells, monocytes, DC cells (Figure 6A). In other words, the decreased expression of *TCF4* and *TFPI* are associated with a low level of immune infiltrations or immune desert in TME. Interestingly, current studies keep the point that a certain of the desert immune microenvironment (DIM) in patients caused by CAFs can result in poor outcomes of anti-PD-1 therapy for patients (11). It is reasonable that *TCF4* and *TFPI* play an important role in inhibiting CAFs-mediated DIM. For instance, *TCF4*, known as immunoglobulin transcription factor 2 (*ITF*-2; *E2-2*; *SEF-2*), contributes to the development of lymphoid (79) and mature plasmacytoid dendritic cells (pDCs), including Pitt-Hopkins syndrome caused by *E2-2* haploinsufficiency (80) and the tumor-suppressive effect in SHH medulloblastoma (81). In all, the inhibition of *TCF4* and *TFPI* expression may lead to CAFs-related desert immune microenvironment, which results in the non-response to anti-PD-1 therapy of patients.

We also analyzed the correlation between six KCDEGs and CAFs-related stimulators, antitumor immune-related molecules as well as immune checkpoint-related molecules. All KCDEGs except *CPXM1* and *GJA1* were positively relevant to most of the mentioned molecules. This suggests that KCDEGs are directly or indirectly involved in the activation process of CAFs and affect the response to immunotherapy in melanoma patients, which is consistent with our previous conclusion that CAFs-related genes can serve as a predictive marker panel of response to anti-PD-1 therapy.

Although the six KCDEGs can distinguish responders and non-responders of anti-PD-1 immunotherapy, the therapeutic efficacy is often influenced by multiple factors in the TME and immune system. Our results also demonstrated an inconsistent correlation of each KCDEGs with immune fractions. This implies the predictive limitation of the accuracy and specificity in anti-PD-1 immunotherapy using a single marker. Combining two or more markers may be more effective for predicting the efficacy of anti-PD-1 therapy (82). To this end, we first employed multivariate Cox regression analysis to assess the OS and TME variety of combined-KCDEGs in SKCM patients. Survival analysis revealed worse OS in the high-risk KCDEGs group. In addition, the two groups had significant differences in multiple TME metrics and anti-tumor immune components. Immune score and cells (including CD8+ naive T cells, DCs, macrophages, and monocytes) tended to decrease in the high-risk group, suggesting that the overall high-risk score of KCDEGs relates to immune infiltration suppression and PD-1 therapy non-response. And this is supported by poorer survival in the high-risk group. Surprisingly, the AUC of combined-KCDEGs (the six-gene panel) was 90.5% in the discovery sets and reached 75.4~100% in the validation sets, which is a substantial improvement compared with the previous study (83) (AUC = 0.75 in the discovery sets and 0.71 in the validation sets). These results fully demonstrate the significant advantage of KCDEGs in anti-PD-1 efficacy prediction. It also indicates that the vital role of CAFs in patient response to anti-tumor immunotherapy may be beyond our imagination and deserves to be explored in detail.

We identified six key genes with predictive value for anti-PD-1 treatment efficacy in melanoma patients via WGCNA and supervised machine learning. However, several limitations remain in this study. We applied robust computational biology methods, including WGCNA, supervised random forest, and LASSO regression, to screen predictive gene panels from multiple perspectives, but validation by experimental biology methods such as RT-PCR is also urgently needed. Secondly, the limited number of samples may make the efficacy assessment model inaccurate, cohorts with small samples often lead to overfitting of machine learning model and reduce the prediction accuracy, thus the model needs to be validated in a larger patient cohort. Thirdly, it is necessary to evaluate the actual predictive effect of the six-gene panel in a clinical patient cohort. Finally, the regulatory mechanism of KCDEGs induced by CAFs in immunotherapeutic suppression is unclear and deserves to explore.

## Conclusion

Using WGCNA combined with supervised machine learning algorithms, we identified a novel CAFs-related panel, including six genes (*CDK14, SYNPO2, TCF4, GJA1, CPXM1, TFPI*), which can distinguish the response of melanoma patients under anti-PD-1 immunotherapy. The multigene may become a potential biomarker panel to guide immunotherapy in the future.

## Data Availability Statement

The datasets presented in this study can be found in online repositories. The names of the repository/repositories and accession number(s) can be found in the article/Supplementary Material.

## Author Contributions

LT and FL contributed to the study inception and design. LT, FL, JL, and YT equally analyzed the data. JC and ML contributed to the study design and study supervision. All authors contributed to the manuscript writing and approved the final version of the manuscript.

## Funding

This study was supported by the Science Innovation Program of College of Laboratory Medicine, Chongqing Medical University (CX201704), the Science and Technology Research Plan Project of Chongqing Education Commission (KJQN202100418), and the Natural Science Foundation of Chongqing (No. cstc2021jcyj-msxm0317).

## Conflict of Interest

The authors declare that the research was conducted in the absence of any commercial or financial relationships that could be construed as a potential conflict of interest.

## Publisher's Note

All claims expressed in this article are solely those of the authors and do not necessarily represent those of their affiliated organizations, or those of the publisher, the editors and the reviewers. Any product that may be evaluated in this article, or claim that may be made by its manufacturer, is not guaranteed or endorsed by the publisher.

## Supplementary Material

Supplementary Figure 1 The flowchart of study design.

Supplementary Figure 2 Expression analysis of KCDEGs.

Supplementary Figure 3 Expression correlation analysis of KCDEGs with CAFs-related stimulating factors and immunotherapy-related molecules.

Supplementary Figure 4 Single-cell analysis from six datasets.

Supplementary Table 1 56 CAFs markers were collected from literature.

## Abbreviations

CAFs: Cancer associated fibroblasts
ICB: Immune checkpoint blockade
TME: Tumor microenvironment
TMB: Tumor mutation burden
MSI: Microsatellite instability
ECM: Extracellular matrix
MCP-1: Monocyte chemotactic protein 1
NK: Natural killer
EMT: Epithelial mesenchymal transition
CTL: Cytotoxic T lymphocytes
TF: Tissue factor
teLEC: tumor exposed-lymphatic endothelial cells
pDCs: plasmacytoid dendritic cells
DEGs: Differentially expressed genes
CDEGs: CAFs-related differentially expressed genes
KCDEGs: Key CAFs-related differentially expressed genes
TCGA-SKCM: TCGA-skin cutaneous melanoma
GEO: Gene Expression Omnibus
WGCNA: Weighted gene co-expression network analysis
LASSO: Least absolute shrinkage and selection operator
GSEA: Gene set enrichment analysis
MAD: Median absolute deviation
TOM: Topological overlap matrix
MEs: Module eigengenes
GO: Gene Ontology
KEGG: Kyoto Encyclopedia of Genes and Genomes
REAC: Reactome
WP: WikiPathways
ROC: receiver operating characteristic
AUC: Area under curve
OS: overall survival
CDK14: Cyclin Dependent Kinase 14
SYNPO2: Synaptopodin 2
TCF4: Transcription Factor 4
TFPI: Tissue Factor Pathway Inhibitor
GJA1: Gap Junction Protein Alpha 1
CPXM1, Carboxypeptidase X: M14 Family Member 1
LIF: LIF Interleukin 6 Family Cytokine
HSF1: Heat Shock Transcription Factor 1
IL6: Interleukin 6
IL1B: Interleukin 1 Beta
CXCL12: C-X-C Motif Chemokine Ligand 12
CXCL10: C-X-C Motif Chemokine Ligand 10
CXCL9: C-X-C Motif Chemokine Ligand 9
CXCR3: C-X-C Motif Chemokine Receptor 3
CCL5: C-C Motif Chemokine Ligand 5
CCL4: C-C Motif Chemokine Ligand 4
TIGIT: T Cell Immunoreceptor With Ig And ITIM Domains
LAG3: Lymphocyte Activating 3
CTLA4: Cytotoxic T-Lymphocyte Associated Protein 4
CD274: CD274 Molecule
PDCD1: Programmed Cell Death 1.
