# Supplementary material for: A Cancer Associated Fibroblasts-Related Six-Gene Panel for Anti-PD-1 Therapy in Melanoma Driven by Weighted Correlation Network Analysis and Supervised Machine Learning
Source: Front Med (Lausanne). 2022 Apr 11;9:880326. doi: 10.3389/fmed.2022.880326 (PMC9035939; doi:10.3389/fmed.2022.880326)
Supplement: Supplementary Figure 1 — The flowchart of study design. [file Data_Sheet_1.DOCX]

Supplementary Material

## Supplementary Figures

**
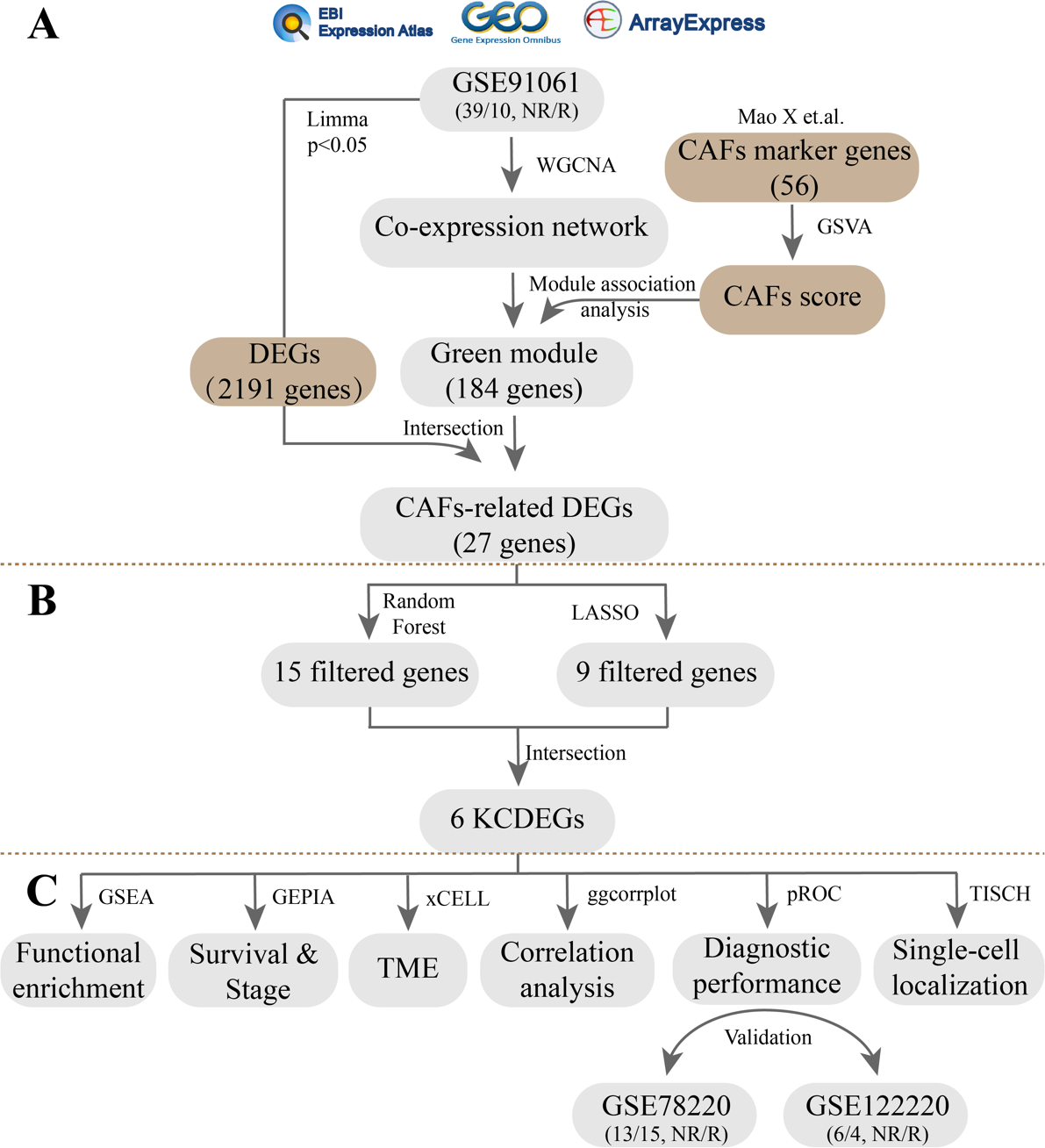
**

**Supplementary Figure 1. The flowchart of study design.**

**(A)** Screening of CAFs-related DEGs by WGCNA. **(B)** Key CAFs-related DEGs extraction based on machine learning models. **(C)** Validation of KCDEGs by multidimensional system analysis. NR: Non-response; R: Response; CAFs: Cancer associated fibroblasts; DEGs: Differentially expressed genes; KCDEGs: Key CAFs-related DEGs; WGCNA: Weighted gene co-expression network analysis; GSVA: Gene set variation analysis; LASSO: Least absolute shrinkage and selection operator; GSEA: Gene set enrichment analysis; GEPIA: Gene expression profiling interactive analysis; TISCH: Tumor immune single cell hub.

**
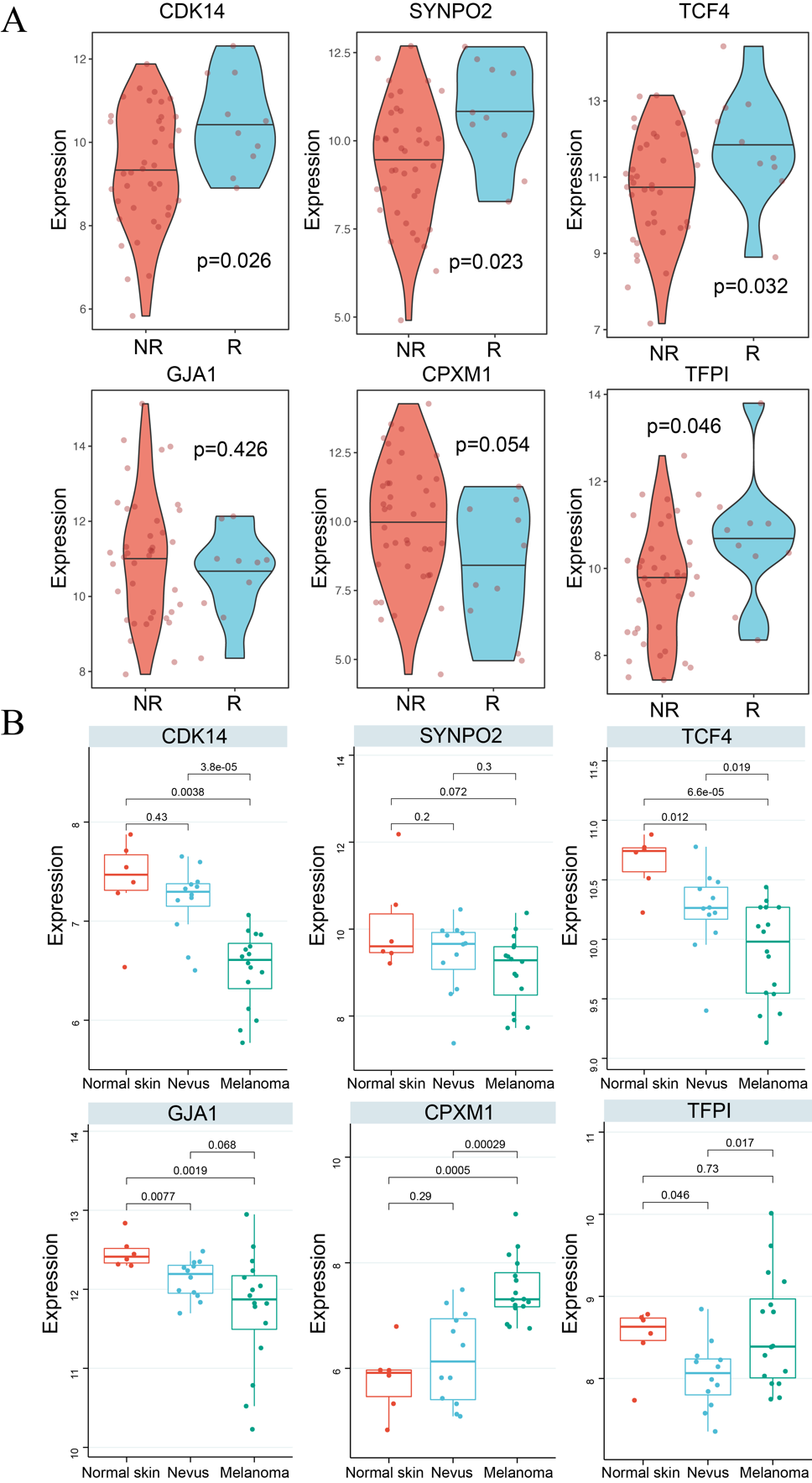
**

**Supplementary Figure 2. Expression analysis of KCDEGs.**

**(A)** Expression of KCDEGs in non-response (NR, n = 39) and response (R, n = 10) to anti-PD-1 therapy in GSE91061. **(B)** Expression of KCDEGs among normal skin (n = 6), nevus (n = 12), and melanoma (n = 16) tissue samples in GSE114445.

**
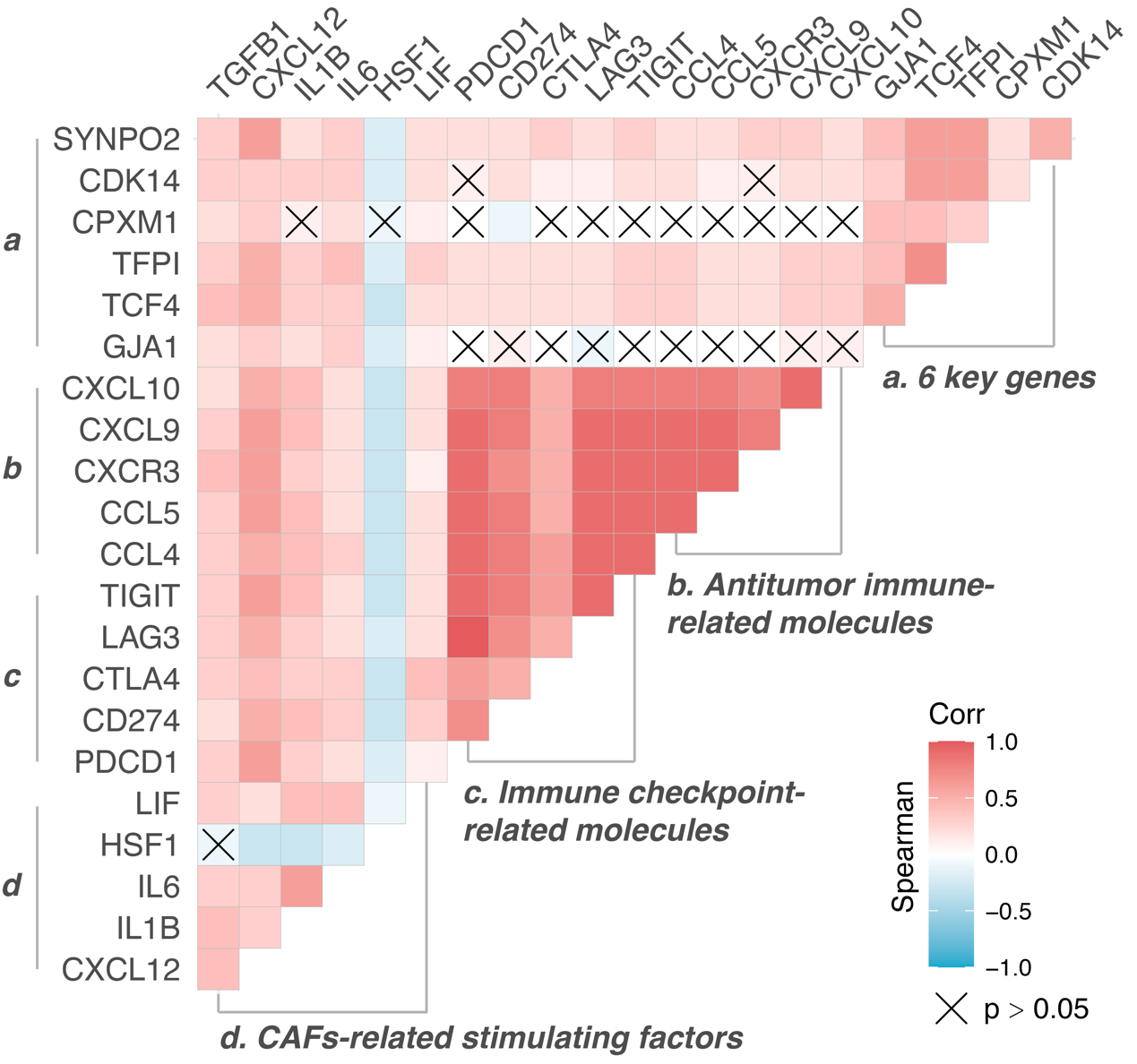
**

**Supplementary Figure 3. Expression correlation analysis of KCDEGs with CAFs-related stimulating factors and immunotherapy-related molecules.** Heatmap showing the Spearman correlation of KCDEGs with CAFs-related stimulating factors, antitumor immune-related molecules, and immune checkpoint-related molecules.

**
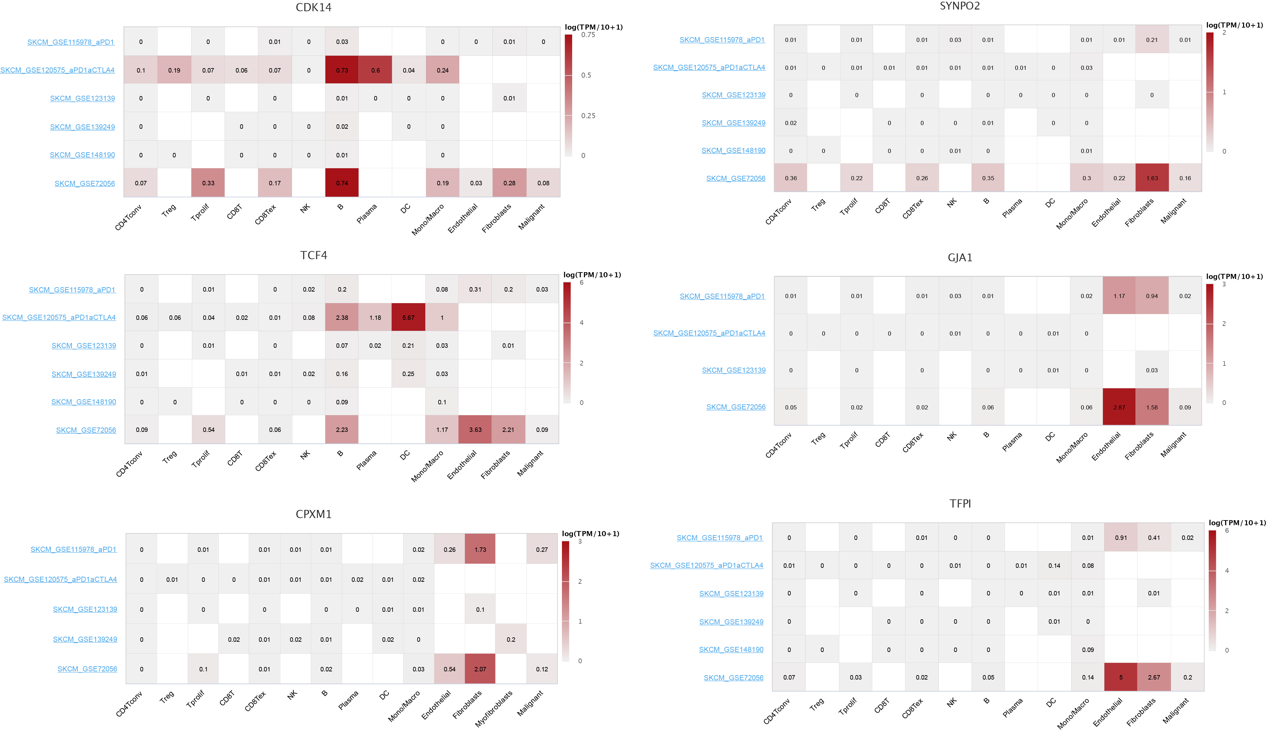
**

**Supplementary Figure 4. Single-cell analysis from six datasets.** Heatmap showing KCDEGs expression of different cell types in six single-cell RNA-seq datasets analyzed by Tumor Immune Single Cell Hub.
